# Identity by descent analysis identifies founder events and links *SOD1* familial and sporadic ALS cases

**DOI:** 10.1038/s41525-020-00139-8

**Published:** 2020-08-07

**Authors:** Lyndal Henden, Natalie A. Twine, Piotr Szul, Emily P. McCann, Garth A. Nicholson, Dominic B. Rowe, Matthew C. Kiernan, Denis C. Bauer, Ian P. Blair, Kelly L. Williams

**Affiliations:** 1 Macquarie University Centre for Motor Neuron Disease Research, Department of Biological Sciences, Faculty of Medicine, Health and Human Sciences, Macquarie University, Sydney, NSW Australia; 2 Transformational Bioinformatics, Commonwealth Scientific and Industrial Research Organisation, Sydney, NSW Australia; 3 Data61, Commonwealth Scientific and Industrial Research Organisation, Dutton Park, QLD Australia; 4 Concord Clinical School, ANZAC Research Institute, Concord Repatriation Hospital, Sydney, NSW Australia; 5 Sydney Medical School, University of Sydney, Sydney, NSW Australia; 6 Department of Clinical Medicine, Faculty of Medicine, Health and Human Sciences, Macquarie University, Sydney, NSW Australia; 7 Brain and Mind Centre, The University of Sydney, Sydney, NSW Australia; 8 Department of Neurology, Royal Prince Alfred Hospital, Sydney, NSW Australia

**Keywords:** Haplotypes, Amyotrophic lateral sclerosis, Mutation

## Abstract

Amyotrophic lateral sclerosis (ALS) is a neurodegenerative disorder characterised by the loss of upper and lower motor neurons resulting in paralysis and eventual death. Approximately 10% of ALS cases have a family history of disease, while the remainder present as apparently sporadic cases. Heritability studies suggest a significant genetic component to sporadic ALS, and although most sporadic cases have an unknown genetic aetiology, some familial ALS mutations have also been found in sporadic cases. This suggests that some sporadic cases may be unrecognised familial cases with reduced disease penetrance in their ancestors. A powerful strategy to uncover a familial link is identity-by-descent (IBD) analysis, which detects genomic regions that have been inherited from a common ancestor. IBD analysis was performed on 83 Australian familial ALS cases from 25 families and three sporadic ALS cases, each of whom carried one of three *SOD1* mutations (p.I114T, p.V149G and p.E101G). We defined five unique 350-SNP haplotypes that carry these mutations in our cohort, indicative of five founder events. This included two founder haplotypes that carry *SOD1* p.I114T; linking familial and sporadic cases. We found that *SOD1* p.E101G arose independently in each family that carries this mutation and linked two families that carry *SOD1* p.V149G. The age of disease onset varied between cases that carried each *SOD1* p.I114T haplotype. Linking families with identical ALS mutations allows for larger sample sizes and increased statistical power to identify putative phenotypic modifiers.

## Introduction

Amyotrophic lateral sclerosis (ALS) is a severe neurodegenerative disorder characterised by the progressive loss of upper and lower motor neurons in the motor cortex, brainstem and spinal cord, resulting in paralysis and death, typically from respiratory failure, within 3–5 years of disease onset^1–5^. The majority of cases present without a family history (sporadic ALS), whereas 5–10% of cases are familial^6^. In most cases, the cause of ALS remains unknown^7^; however, heritability studies suggest a significant genetic component to sporadic ALS^8,9^. Genetic mutations that are present in familial ALS cases have also been found in sporadic ALS cases^10,11^, suggesting that some sporadic cases may in fact be unrecognised familial cases, perhaps due to reduced disease penetrance in their ancestors. Identifying a familial basis of disease in apparently sporadic ALS cases has important genetic counselling implications for their immediate family members, including a 50% chance of inheriting the mutation and an increased likelihood of developing ALS.

Mutations in the gene encoding copper zinc superoxide dismutase 1 (*SOD1* [NM_000454, NP_000445]) account for around 20% of familial ALS cases^2,3,5^ and a small proportion of sporadic ALS cases^10,12^. More than 150 mutations in *SOD1* have been associated with ALS thus far, where the frequency of each mutation varies across populations. The most common *SOD1* mutation in North America is p.A5V (c.14C>T), whereas in Scandinavia and the United Kingdom the most common *SOD1* mutations are p.D91A (c.272A>C) and p.I114T (c.341T>C), respectively. All three of these *SOD1* mutations, as well as *SOD1* p.D12Y (c.34G>T) and p.R116G (c.346C>G), originated from founder events where the mutation descended from a common ancestor^11,13–17^.

Mutations that originate from founder events are typically inherited as part of larger founder haplotypes that are broken down over time due to recombination. In North America, *SOD1* p.A5V is found most often on a haplotype background that suggests it arose in American Indians. In contrast, *SOD1* p.A5V is found on a different haplotype background in Europeans. This indicates two separate founder events for *SOD1* p.A5V^13^. In addition, *SOD1* p.D91A arose from a single founder in Scandinavian families with recessive ALS, while multiple founders exist when this mutation is inherited in a dominant fashion^11,14^. Much of the work on founder events in ALS has used microsatellite markers to identify a founder haplotype^10,11,14,16,17^. However, alternative methods are available that make use of tens-of-thousands of single nucleotide polymorphisms (SNPs) extracted from SNP array data or whole-genome sequencing (WGS) data, which can also provide fine-scale resolution on the breakpoints of shared ancestral haplotypes and more accurate variant dating. These methods identify genomic regions that have been inherited from a recent common ancestor, said to be identical by descent (IBD), and have proven useful in many applications, including disease mapping^18,19^ and uncovering unknown relatedness^20,21^. In the case of founder events, individuals who have inherited part of a founder haplotype are in fact IBD over this genomic region, therefore inferred IBD regions can be used to identify common founders and thus founder events^19^.

In this study, we performed an IBD analysis leveraging WGS data to investigate founder events in a cohort of 83 Australian familial ALS cases from 25 families and three sporadic ALS cases with the most common *SOD1* mutations in Australia: *SOD1* p.I114T (c.341T>C), p.V149G (c.446T>G), p.E101G (c.302A>G)^22^. We identified multiple families and sporadic cases as distantly related and discovered several founder events in patients carrying identical *SOD1* mutations. In particular, we created relatedness networks to visualise clusters of individuals sharing a common haplotype over *SOD1*, from which we subsequently inferred the number of unique haplotype backgrounds that carry each causal *SOD1* mutation in the population, thus drawing conclusions as to the presence of founder events. This suggested that *SOD1* p.I114T and p.E101G each had two independent origins in this cohort, and p.V149G had a single origin; totalling five independent founder events. For each of the five founder events, we defined a unique 350-SNP founder haplotype over *SOD1* on chromosome 21 (481 kb). These haplotypes differentiate between the founder events, providing evidence of independent origins for each mutation. We identified differences in the age of onset and rate of disease progression between cases that carried an identical *SOD1* p.I114T or p.E101G mutation on different haplotype backgrounds. Furthermore, we were able to calculate the time to the most recent common ancestors for both *SOD1* p.I114T and p.V149G as <360 years ago.

## Results

### Summary statistics for the *SOD1* cohort

Following filtering procedures of WGS data, 86 ALS samples and 3,527,233 SNPs genome wide were retained for analysis. Of these, 83 cases had familial ALS, where 41 individuals (21 families) carry a *SOD1* [NM_000454.4] p.I114T (c.341T>C) mutation, 33 individuals (two families) carry *SOD1* p.V149G (c.446T>G), and nine individuals (two families) carry *SOD1* p.E101G (c.302A>G) (Table 1). In addition, routine mutation screen of *SOD1* identified three sporadic ALS cases with a *SOD1* p.I114T mutation^23^. Pairwise IBD analysis was performed on the SNP data using TRIBES^24^. A total of 16,414 IBD segments of 3 cM or greater were retained genome-wide and used to estimate the degree of relatedness between pairs of cases, while 1204 IBD segments of 3 cM or greater on chromosome 21 were used for network analysis and haplotype construction.

**Table 1:** Familial and sporadic ALS *SOD1* mutation carrier samples.

Familial or sporadic	Family or sporadic ID	Number of samples	Number of pairs^a^	*SOD1* mutation
Sporadic	SALS	3	–	p.I114T
Familial	3	6	15	p.E101G
Familial	12	15	105	p.I114T
Familial	18	32^b^	496	p.V149G
Familial	35	1	0	p.V149G
Familial	43	2	1	p.I114T
Familial	76	1	0	p.I114T
Familial	95	2	1	p.I114T
Familial	98	1	0	p.I114T
Familial	106	1	0	p.I114T
Familial	123	1	0	p.I114T
Familial	124	2	1	p.I114T
Familial	130	1	0	p.I114T
Familial	131	1	0	p.I114T
Familial	132	3	3	p.E101G
Familial	197	2	1	p.I114T
Familial	213	1	0	p.I114T
Familial	259	1	0	p.I114T
Familial	267	1	0	p.I114T
Familial	270	1	0	p.I114T
Familial	274	1	0	p.I114T
Familial	286	1	0	p.I114T
Familial	334	2	1	p.I114T
Familial	374	2	1	p.I114T
Familial	mq10	1	0	p.I114T
Familial	mq36	1	0	p.I114T
Total	–	86	625	–

^a^The number of pairwise comparisons between samples was calculated for familial samples only and was simply the number of unordered 2-sample combinations, i.e. *n*-choose-2, where *n* was the number of samples.

^b^WGS data from five additional samples did not pass WGS-processing quality thresholds and were not used in subsequent analyses.

### New relationships identified between ALS families and sporadic cases

**Fig. 1:**
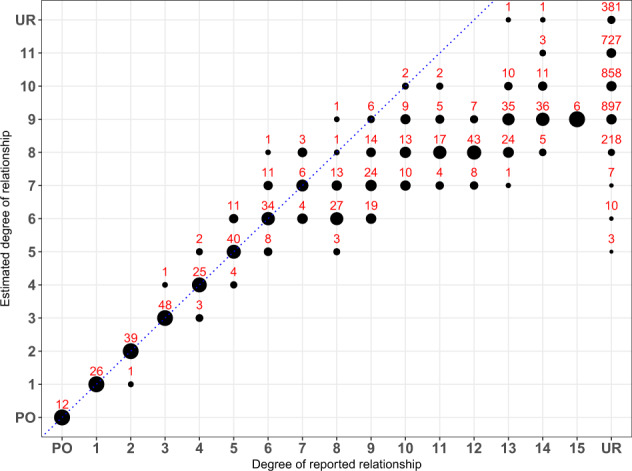
The reported vs. estimated degree of relatedness in the *SOD1* cohort using TRIBES. The size of the circles represent the percentage of individual pairs whose estimated degree of relationship are exactly the same as their reported relationship. The number of pairs estimated at each point is labelled above the corresponding circle. PO and UR are abbreviations for parent–offspring pairs and unrelated pairs, respectively. Individuals were reported as unrelated if they belonged to different families or were sporadic cases. Circles that fall on the dotted line, *y* = *x*, indicate concordance between the reported and estimated relationship. TRIBES correctly estimated 99% of relationships to within 1 degree of the reported relationships for relatives up to 7th degree (third cousins) and identified 3, 10 and 7 pairs of seemingly unrelated individuals as 5th, 6th and 7th degree relatives, respectively. Degrees of reported relationships are obtained by the value of the kinship coefficients given a pedigree.

Of the 83 familial ALS cases, 68 came from families where multiple affected individuals were sequenced and the degree of relatedness was known (Table 1). Of these known relationships, TRIBES correctly estimated 99% of relationships to within 1 degree of the true relationship for relatives up to 7th degree (third cousins), while only 13% of 8th degree or higher relatives were correctly estimated to within 1 degree (Fig. 1).

By extending this analysis to identify relationships between seemingly unrelated individuals, 3, 10 and 7 pairs of individuals were found to be 5th, 6th and 7th degree relatives, respectively (Fig. 1; Table 2), while there were no individuals of unknown relatedness who were estimated as 4th degree relatives or closer. Although some apparently unrelated individuals were inferred as 8th to 11th degree relatives (Fig. 1), we chose only to investigate individuals identified as 7th degree relatives or closer as this is the accuracy limit of TRIBES^24^. Of these novel relationships, 19 pairs were from patients where both individuals within each pair had identical *SOD1* variants and shared an IBD segment over this locus. This included one pair of apparently sporadic ALS cases (MN201517 and SALS2258) with *SOD1* variants, which confirmed they are in fact part of a larger extended family.

**Table 2:** Newly identified 5th, 6th and 7th degree-related pairs.

FID^a^ 1	IID^b^ 1	FID^a^ 2	IID^b^ 2	Estimated degree	IID 1 mutation	IID 2 mutation
18	18-60	35	35-142	5	p.V149G	p.V149G
18	18-58	35	35-142	5	p.V149G	p.V149G
197	197-060095	mq36	mq36-MQ160147	5	p.I114T	p.I114T
18	18-77	35	35-142	6	p.V149G	p.V149G
18	18-67	35	35-142	6	p.V149G	p.V149G
18	18-77	197	197-060228	6	p.V149G	p.I114T
334	334-060820	374	374-140839	6	p.I114T	p.I114T
334	334-120512	374	374-140839	6	p.I114T	p.I114T
334	334-060820	374	374-140975	6	p.I114T	p.I114T
334	334-120512	374	374-140975	6	p.I114T	p.I114T
123	123-971530	259	259-080285	6	p.I114T	p.I114T
197	197-060228	mq36	mq36-MQ160147	6	p.I114T	p.I114T
SALS	MN201517^c^	SALS	SALS2258^c^	6	p.I114T	p.I114T
76	76-940290	SALS	MN201517	7	p.I114T	p.I114T
76	76-940290	SALS	SALS2258	7	p.I114T	p.I114T
267	267-090221	286	286-090750	7	p.I114T	p.I114T
18	18-41	35	35-142	7	p.V149G	p.V149G
197	197-060095	43	43-070626	7	p.I114T	p.I114T
197	197-060095	43	43-080797	7	p.I114T	p.I114T
197	197-060228	43	43-080797	7	p.I114T	p.I114T

^a^Family or sporadic ID.

^b^Individual ID.

^c^Two sporadic cases were inferred as close relatives (MN201517 and SALS2258). The third sporadic case was inferred as an 8th degree relative or high with other *SOD1* p.I114T carries, thus is not present in this table.

### Identification of five independent *SOD1* mutation founder events

**Fig. 2:**
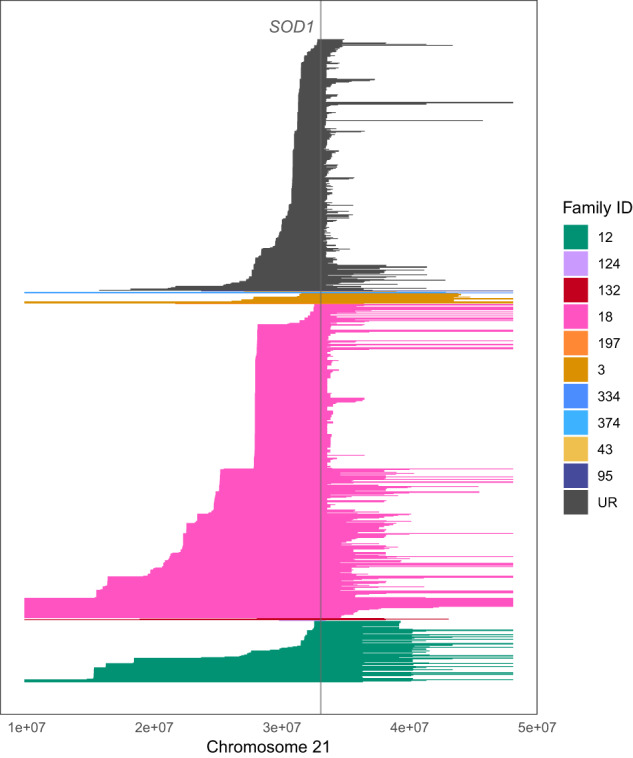
The distribution of IBD segments that overlap *SOD1*. Each line represents an IBD segment inferred between a unique pair. IBD segments have been coloured according to whether both individuals within a pair belong to the same family; or whether they belong to different families and are otherwise considered unrelated (UR). All three sporadic ALS patients with *SOD1* variants were considered unrelated. Family 18 had the greatest number of IBD segments inferred over *SOD1* as this family had the greatest number of cases sequenced, followed by family 12. Many IBD segments were inferred over *SOD1* between apparently unrelated individuals, suggesting these individuals were part of an extended family.

Of all individuals with *SOD1* mutations, IBD segments over the *SOD1* locus were expected in 625 pairs since this was the total number of pairs known to be related prior to analysis (Table 1). However, there was more IBD sharing over *SOD1* than expected (Fig. 2). We observed IBD segments in 954 pairs that indicated shared haplotypes between seemingly unrelated families and sporadic cases, where the median length of an IBD segment over *SOD1* in apparently unrelated individuals was 4 cM (range: 3–37.69 cM).

**Fig. 3:**
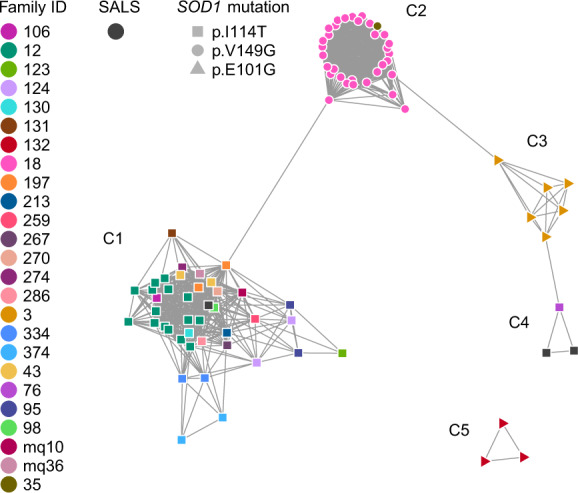
Network of individuals sharing IBD segments over *SOD1*. Each node is a sample and an edge was drawn between two samples if they were inferred IBD over *SOD1*. Nodes are positioned according to the Fruchterman–Reingold force-directed layout^39^, where there is no meaning behind the edge lengths. Nodes are coloured according to their unique family ID, in addition to the three sporadic ALS cases who have been assigned one colour. All samples had one of three *SOD1* mutations, represented by unique node shapes in the network. There are five clusters in this network, denoted C1–C5, where all cases within each cluster had an identical *SOD1* mutation. Cluster C2, where individuals carried *SOD1* p.V149G (c.446T>G), connects family 18 and family 35, indicating they were in fact one family. Similarly, two clusters are present for individuals that carried *SOD1* p.I114T (c.341T>C) (clusters C1 and C4), where these individuals were from different families, including three apparently sporadic ALS cases, indicating two disjoint extended families. Specifically, two sporadic cases were found to be related to each other and family 76 (cluster C4), whereas the third sporadic case was found to be related to the remaining 20 families with *SOD1* p.I114T (cluster C1). In contrast, *SOD1* p.E101G (c.302A>G) was unique to each family with this mutation (clusters C3 and C5), suggesting independent origins. The three pairs of individuals with discordant mutations who were inferred IBD over *SOD1* did not share the disease-associated haplotypes and likely represent false-positive IBD calls.

A relatedness network of individuals that shared IBD segments over *SOD1* is shown in Fig. 3. Noticeably, five distinct clusters were evident, where every individual within each cluster carried the same *SOD1* mutation on identical haplotype backgrounds. A unique 350-SNP founder haplotype overlapping *SOD1* was extracted for each of the five clusters (Supplementary Data 1 and Supplementary Fig. 1). The 481 kb (0.56 cM) interval over which the founder haplotypes are reported (hg19—chr21:32,792,891–33,274,026) denotes the intersection of SNPs common to all five founder haplotypes and can accurately distinguish between each founder event (Supplementary Data 1 and Supplementary Fig. 1).

**Fig. 4:**
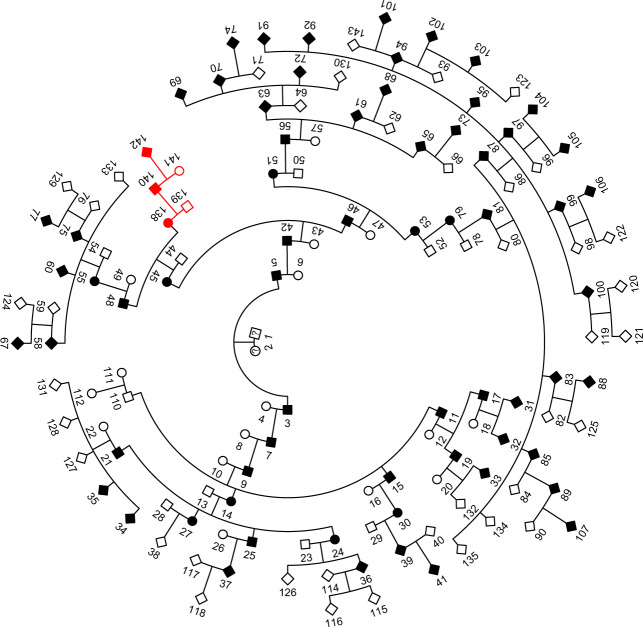
Pedigree connecting two Australian families with *SOD1* p.V149G. A subset of family 18’s pedigree with 67 ALS cases over ten generations linked to family 35 (individual IDs 138–142). The extended pedigree for family 18 has 409 individuals and 67 ALS cases. The sex of individuals from generation 7–10 have been omitted for confidentiality.

Both families with the *SOD1* p.V149G mutation shared a common haplotype over this locus (cluster C2 in Fig. 3, Supplementary Data 1), which suggests that p.V149G descended from a common founder. Relationship estimates between cases from each family identified two pairs of 5th degree relatives as well as more distant relatives linking both families (Table 2 and Fig. 4). In contrast, *SOD1* p.E101G was found on two different haplotype backgrounds (clusters C3 and C5 in Fig. 3, Supplementary Data 1, Supplementary Fig. 1), each unique to one of the two families that carried this mutation, suggesting that p.E101G arose independently in these families. Cases that carried *SOD1* p.E101G in cluster C5 typically presented with ALS six years earlier than cases in cluster C3 and had a more rapid disease progression (mean of 3.8 years vs. 10 years from disease onset until death, Table 3). Only a small number of samples from each cluster have complete clinical data, as such it is not possible to assess the statistical significance of the difference in age of disease onset and the rate of disease progression between clusters C3 and C5. Similarly, two different haplotype backgrounds harbour the *SOD1* p.I114T mutation (clusters C1 and C4 in Fig. 3, Supplementary Data 1, Supplementary Fig. 1), implying two independent origins for this mutation in our cohort. The less common haplotype was seen in three cases (cluster C4); including two apparently sporadic cases and one familial case. These three individuals were estimated to be 6th and 7th degree relatives. The more common *SOD1* p.I114T haplotype was present in 20 apparently unrelated families as well as one apparently sporadic case (cluster C1), suggesting this haplotype had also descended from a common founder and was the most widely distributed haplotype in our cohort. The closest degree of relatedness estimated between families in this cluster was 5th degree (Table 2). While a sex bias in ALS typically results in one-third more males being affected with the disease, there were one-third more females affected in this Australian cohort who carried the *SOD1* p.I114T mutation (Table 3). Moreover, cases that carried *SOD1* p.I114T in cluster C4 typically presented with ALS 16 years earlier (mean age 40.8 years) than cases that carried *SOD1* p.I114T on the more common haplotype in cluster C1. Due to incomplete clinical data for cases in clusters C1 and C4, we could only assess trends, instead of statistical differences, in ages of disease onset between clusters.

**Table 3:** Summary of Australian ALS clinical information for each of the five *SOD1* clusters.

Cluster	*SOD1* mutation	No. of mutation carriers	% Male	% Spinal [*n*]	Mean age (years) at onset ± SD (range) [*n*]	Mean age (years) at death ± SD (range) [*n*]	Mean disease duration (months) ± SD (range) [*n*]
C1	p.I114T	41	41%	94% [16]	57.1 ± 14.5 (29.5–76.0) [16]	61.5 ± 12.6 (35.1–78.2) [18]	64.1 ± 45.7 (28.5–177.1) [11]
C4	p.I114T	3	33%	100% [3]	40.8 ± 12.5 (30.2–54.5) [3]	NA	NA
C2	p.V149G	33	60%	75% [16]	43.0 ± 8.7 (25.1–57.6) [17]	44.8 ± 7.8 (25.8–53.8) [21]	29.5 ± 34.3 (7.8–145.0) [16]
C3	p.E101G	6	50%	100% [4]	48.6 ± 9.9 (36.2–60.2) [4]	64.2 ± 15.2 (45.9–83.0) [4]	119.8 ± 59.1 (62.8–180.9) [3]
C5	p.E101G	3	67%	50% [2]	42.6 [1]	51.7 ± 11.7 (43.6–65.1) [3]	46.0 [1]

Number of cases with available clinical data is indicated by square brackets.

*SD* standard deviation.

### Mutation dating of *SOD1* p.V149G and p.I114T

We estimated the times to the most recent common ancestor for *SOD1* p.V149G and p.I114T, where estimation was performed separately for each of the two clusters that carried p.I114T (Fig. 3). For *SOD1* p.V149G, we selected six individuals for analysis, including individuals from both families, who were at least 6th degree relatives. The estimated age of p.V149G was 3–11 generations (60–220 years, assuming 20-year generation time). For *SOD1* p.I114T cluster C1, we selected one individual from each of the 20 families with the highest number of connections to other individuals in the network as well as the sporadic case for variant dating. The estimated age of p.I114T on the haplotype present in this cluster was between 5 and 18 generations (100–360 years). For *SOD1* p.I114T cluster C4, we included all three individuals in the calculation, and estimated the age of p.I114T on the alternative haplotype to be between 1 and 11 generations (20–220 years).

## Discussion

In the present study, we analysed a cohort of Australian ALS cases with causal mutation identified as *SOD1* p.I114T, p.V149G and p.E101G^22,23^. However, as each of these three mutations appeared in multiple individuals from different families, we sought to determine if each mutation descended from one or more common ancestor. In the case of *SOD1* p.I114T, where 41 individuals from 21 families and three sporadic cases had the mutant allele, it seemed unlikely that this mutation arose independently in each family, reflecting a high mutation rate. As such, we performed an IBD analysis on WGS data to uncover any unknown relatedness in this cohort and explore founder events.

Using TRIBES to estimate the degree of relatedness between apparently unrelated individuals, we identified 20 pairs of 5th, 6th and 7th degree relatives connecting six pairs of families, where both individuals had identical *SOD1* mutations in all but one pair. Investigating the pair with discordant mutations revealed the inferred IBD segments to be inconsistent with Mendelian inheritance (data not shown), thus they are unlikely to represent true 6th degree relatives. One explanation for incorrectly identifying these individuals as close relatives is the increased number of false IBD segments produced by GERMLINE with sequencing data^25^. Many incorrectly inferred IBD segments will inflate the amount of IBD sharing observed between a pair of individuals, which in turn will give the appearance of close relatives. This may also explain why more distant relatives, such as individuals who are 12th degree relatives or greater, are consistently estimated as more closely related (Fig. 1).

Relatedness networks have been shown to be a powerful method to identify clusters of individuals sharing a common haplotype over a locus and can also be informative as to the number of haplotypes that segregate with disease, indicative of independent origins or founder events^19,26^. By investigating IBD segments overlapping *SOD1* using relatedness networks, we identified five distinct clusters of individuals that each carried a unique 350-SNP disease-associated haplotype (Fig. 3; Supplementary Data 1 and Supplementary Fig. 1). Multigenerational pedigrees were available in four of the five clusters of related individuals identified over *SOD1*, thus risk haplotypes were observed as segregating with disease in these families. Biallelic SNPs provided in Supplementary Data 1 allow for experimental validation of haplotypes in additional *SOD1* cohorts. Three of these clusters were each connected by one pair of individuals with discordant *SOD1* mutations, whom are unlikely to be truly related. *SOD1* p.I114T was present on two different haplotype backgrounds, one of which was inherited in 20 families and one sporadic case. p.I114T is the most common *SOD1* mutation in the United Kingdom, and in particular in Scotland^15^, where haplotype analysis of Scottish p.I114T mutant cases revealed a common founder^10,27^. It is likely that *SOD1* p.I114T in the Australian cohort has also descended from Scottish founders, as genealogical analysis indicated that six of the p.I114T families originated from Scotland, including families in both clusters that carry different *SOD1* p.I114T haplotypes (Fig. 3). Furthermore, we estimated that this mutation originated from a common ancestor up to 360 years ago, which is within the timeframe of Scottish settlers in Australia^28^.

Family 18 was the largest Australian ALS family in the cohort, spanning ten generations, 409 total individuals and 67 ALS cases with the *SOD1* p.V149G mutation^29^, of which 32 were included in this analysis. TRIBES inferred two individuals from family 18 as both 5th and 6th degree relatives with a single case from family 35, who also carried a *SOD1* p.V149G mutant allele. Using the relationship estimates from TRIBES along with pedigree records, we were able to create a new pedigree combining both families (Fig. 4). Relationship estimates combined with the inferred IBD segments confirmed that all cases with p.V149G in this cohort descended from a common founder; predicted to have originated up to 11 generations ago (220 years), which was consistent with pedigree records.

Cases that carried the *SOD1* p.I114T disease haplotype in cluster C4 typically presented with ALS 16 years earlier than cases in cluster C1. Moreover, cases that carried the *SOD1* p.E101G disease haplotype in cluster C5 also presented with disease earlier and had a more rapid disease progression than cases with an identical *SOD1* mutation in cluster C3. Given the small number of cases in these clusters, along with incomplete clinical records, more samples are required to evaluate the statistical significance of these findings. However, our initial results presented here suggest that clustering ALS cases based on disease haplotypes may be useful for prognostic testing, which would better prepare clinicians for the needs and care of their patients and be invaluable for patients and their families.

The *SOD1* mutations described here have historically been considered to have a large effect size^6^ since they are predominantly identified in familial ALS cases, highly penetrant in affected families and almost always present as classic ALS without comorbid frontotemporal dementia. Yet we, and others, have shown that *SOD1* mutations can also be present in apparently sporadic ALS cases^23^. The variability observed in disease phenotype, including age of disease presentation and duration, between individuals carrying identical mutations, suggests polygenic, epigenetic and environmental factors may also have a role in disease onset and progression^23^. This was evident in this Australian cohort where the age of disease onset differed between cases that carried each *SOD1* p.I114T haplotype, whereas the rate of disease progression differed between cases that carried each *SOD1* p.E101G haplotype. It has been postulated that separating ALS into phenotype subgroups may aid in uncovering phenotypic modifiers, whether they be genetic or epigenetic. Large ALS families with known gene mutations provide a relatively homogenous group with which to uncover modifiers. However, the late onset of ALS limits the recruitment of affected individuals, such that most recruited ALS families are represented by a small number of samples. By genetically linking families using relatedness analysis, specifically IBD sharing, we can increase family sizes and therefore increase statistical power to identify phenotypic modifiers.

Phenotypic modifiers may also explain why some ALS cases appear as sporadic cases when they are in fact familial cases with reduced disease penetrance in their ancestors. Here, all three apparently sporadic ALS cases that carried a *SOD1* p.I114T mutation were shown to be unrecognised familial cases. This result is consistent with previous findings that familial ALS cases with *SOD1* p.I114T have been incorrectly classified as sporadic cases^10,27^. Additional variants may be acting as disease modifiers or to reduce disease penetrance. In addition to incomplete penetrance, incorrect classification of sporadic ALS cases may arise from inadequate knowledge or reporting of family history and may be masked, for example, by the death of at-risk family members from other causes prior to ALS onset^6,30^. Not recognising a familial basis of disease can have significant genetic counselling implications for immediate family members^6,30^ whose risk of developing ALS greatly increases. Correct classification of familial and sporadic cases allows health professionals to make appropriate recommendations regarding genetic testing and counselling of ALS patients and their families.

Identifying relatedness and thus founder events within ALS patient cohorts aids in disease gene mapping when the causal variant is unknown. In such instances, the search space for potential candidate genes can be greatly reduced to those within IBD regions common to all affected family members. Such analyses may help improve our understanding of the biological mechanisms influencing familial ALS, particularly in terms of disease progression, as well as sporadic ALS, which remains largely unsolved.

## Methods

### Australian sample cohort

850 Australian participants were recruited for analysis from the Macquarie University Neurodegenerative Disease Biobank, Molecular Medicine Laboratory (Concord Hospital), Australian MND DNA Bank (Royal Prince Alfred Hospital) and Brain and Mind Centre (University of Sydney). Each participant provided informed written consent as approved by the human research ethics committees of the Sydney South West Area Health Service, Macquarie University (5201600387), or University of Sydney. Most participants were of European descent, and each ALS case was clinically diagnosed according to El Escorial criteria^31^. Genomic DNA extraction was performed from whole blood according to standard protocols.

### Whole-genome sequencing data processing

A total of 850 DNA samples from 638 sporadic ALS patients, 103 familial ALS patients and 109 frontotemporal dementia (FTD) patients underwent library preparation using the TruSeq PCR free library preparation kit (Illumina, v2.5). Prepared libraries underwent multiplex 150 bp paired-end sequencing on an Illumina HiSeq X Ten instrument (Kinghorn Centre for Clinical Genomics, Sydney, Australia). Samples were demultiplexed using bcl2fastq (v2.16.0) and aligned against the human reference hs37d5x using BWA-MEM^32^ (v0.7.15-r1140). The mean per-base coverage obtained after alignment of reads to the hg19 reference genome was 40× (range 30×– 47×). Following alignment, bamsormadup (v2.0.65) was used to sort the read alignments, mark duplicate reads and index the BAM files. Local realignment and base quality score recalibration were performed using GATK^33^ (v3.7-0 gcfedb67) IndelRealigner and BaseRecalibrator, respectively. Per-sample BAM files were then merged using sambama merge (v0.6.5) and GATK^33^ HaplotypeCaller was used to call variants and produce the final per-sample Genomic VCF file (gVCF). gVCF files were merged using GATK CombineGVCFs and input into GATK GenotypeGVCFs to call genotype for the complete cohort of samples. Variant recalibration was performed using GATK VariantRecalibrator and a single joint-called VCF file was generated using GATK ApplyRecalibration. Variants in the final joint VCF file were annotated using ANNOVAR^34^ (v2017Jul16).

Samples from the joint VCF file were excluded if they did not pass a number of quality control criteria. The human–human contamination rate was estimated using VerifyBamID (v1.1.3) and samples with a contamination rate >0.03 were excluded. Principle component analysis was used to identify samples from similar population background. Most samples were of European descent and clustered with the two European HapMap Phase 3 populations (CEU, TSI). Samples which did not cluster with these populations were excluded. Samples which had a high level (>5%) of missing genotypes were also filtered out. One sample whose autopsy result came back negative for ALS was also removed. Duplicate samples and unaffected individuals were also removed. A total of 815 samples remained following filtering of the WGS data, including 83 familial ALS cases from 25 families who were previously known to carry either a *SOD1* [NM_000454.4, NP_000445.1] p.I114T (c.341T>C), p.V149G (c.446T>G) or p.E101G (c.302A>G) mutation^22^. Routine mutation screening of *SOD1* in the remaining cases determined that three sporadic ALS cases have a *SOD1* p.I114T mutation^23^. A total of 86 *SOD1* mutations carries (Table 1) and 3,527,233 high quality SNPs remained for analysis.

### IBD analysis

Relationship estimates and IBD segments were inferred using TRIBES^24^ with default parameter settings. Briefly, TRIBES phases biallelic SNP data using BEAGLE v4.1^35^ then infers IBD segments with the phased haplotype data using GERMLINE v1.5.3^36^. GERMLINE identifies IBD segments by sliding a window of a predefined length along a chromosome and classifying pairs of samples as IBD within each window if they have an identical haplotype. Neighbouring windows that are inferred IBD for a pair of samples are then merged to define the IBD segment boundaries. IBD segments that overlapped the masked regions reported in TRIBES were either removed from further analyses or had their boundaries adjusted. These masked regions most likely reflect population substructure due to linkage disequilibrium and loci that are difficult to map such as centromeres^37^. We note that *SOD1* was more than 12 Mb from its nearest masked region. IBD segments of 3 cM or larger (*n* = 16,414) were retained for analysis genome wide. This typically reflects the sizes of IBD segments in recent common ancestors (up to 17 generations or 340 years, assuming 20-year generation time) that can be detected with high power and accuracy^38^, and is within the timeframe of the first migrants to Australia.

### Relatedness networks of shared haplotypes over the *SOD1* locus

A relatedness network is a graphical representation of shared haplotypes between pairs of individuals over a specified locus. Each node in the network represents a unique individual and an edge is drawn between two nodes if the individuals share an IBD segment, either partially or completely, over the specified locus. All individuals who do not share an IBD segment over the locus with any other individual are omitted from the network. Networks are produced using the functions getIBDiclusters and plotIBDclusters in the R package isoRelate^26^, where the network layout is produced according to Fruchterman–Reingold forced-directed layout algorithm^39^. This algorithm aims to position nodes such that all edges are of similar lengths with as few edges overlapping as possible. The locus used in this study was chr21:33,031,935–33,041,243 (hg19).

### Extracting founder haplotypes over *SOD1*

The process for extracting founder haplotypes over the *SOD1* locus on chromosome 21 is illustrated in Supplementary Fig. 2, and R code is provided in Supplementary Data 2. Individuals who form a cluster in the relatedness network share an identical founder haplotype over *SOD1*. The endpoints of the founder haplotype for a cluster are taken as the intersection of the IBD segments inferred between all pairs of individuals within this cluster. Since the genotype data has been phased, the founder haplotype is the singular haplotype that appears in all samples within this cluster between the identified haplotype endpoints. Once the founder haplotype has been generated for each cluster in the relatedness network, the interval over which all of these haplotypes overlap is defined, and each founder haplotype is reported within this interval. Founder haplotypes are reported over the same interval, and hence the same biallelic SNPs, to enable simple and direct comparisons between them. SNPs with an identical allele across all founder haplotypes were removed from the reported haplotypes since they are not informative for the purpose of founder haplotype comparisons.

### Dating *SOD1* mutations p.I114T and p.V149G

The Gamma method^40^ was used to estimate the age of *SOD1* p.I114T and p.V149G, respectively. Variant dating could not be performed on *SOD1* p.E101G as there were too few individuals of sufficiently distant relatedness for the assumptions of the methodology to hold. Briefly, the Gamma method uses the lengths of shared ancestral haplotypes that carry the mutation to estimate the time to the most recent common ancestor, which is less than or equal to the time since the mutation first arose. Ancestral haplotype lengths were simply taken as the lengths of the inferred IBD segments generated from phased data, and the time to the most recent common ancestor is reported assuming a correlated genealogy, which takes into account subsets of samples with a common ancestor earlier than the most recent common ancestor for all samples.

### Reporting summary

Further information on research design is available in the Nature Research Reporting Summary linked to this article.

## Supplementary information

Supplementary Information

Supplementary Data

Reporting Summary

## Data Availability

The genotype and clinical phenotype data that support the findings of this study are not publicly available due to ethics and patient consent constraints. However, genotype and basic clinical phenotype data are available on reasonable request from the corresponding author [K.L.W.] under a collaboration and data usage agreement.

## Code availability

The R code used to extract the founder haplotypes over *SOD1* has been included in the Supplementary Information file (Supplementary Data 2).
