# Supplementary material for: Identity by descent analysis identifies founder events and links SOD1 familial and sporadic ALS cases
Source: NPJ Genom Med. 2020 Aug 7;5:32. doi: 10.1038/s41525-020-00139-8 (PMC7414871; doi:10.1038/s41525-020-00139-8)
Supplement: Supplementary file 1 — Supplementary Information [file 41525_2020_139_MOESM1_ESM.pdf]

Supplementary Information

Identity by descent analysis identifies founder events and links *SOD1* familial and sporadic ALS cases

Lyndal Henden, Natalie A. Twine, Piotr Szul, Emily P McCann, Garth A Nicholson, Dominic B Rowe, Matthew C Kiernan, Denis C Bauer, Ian P Blair and Kelly L. Williams

Supplementary Figures

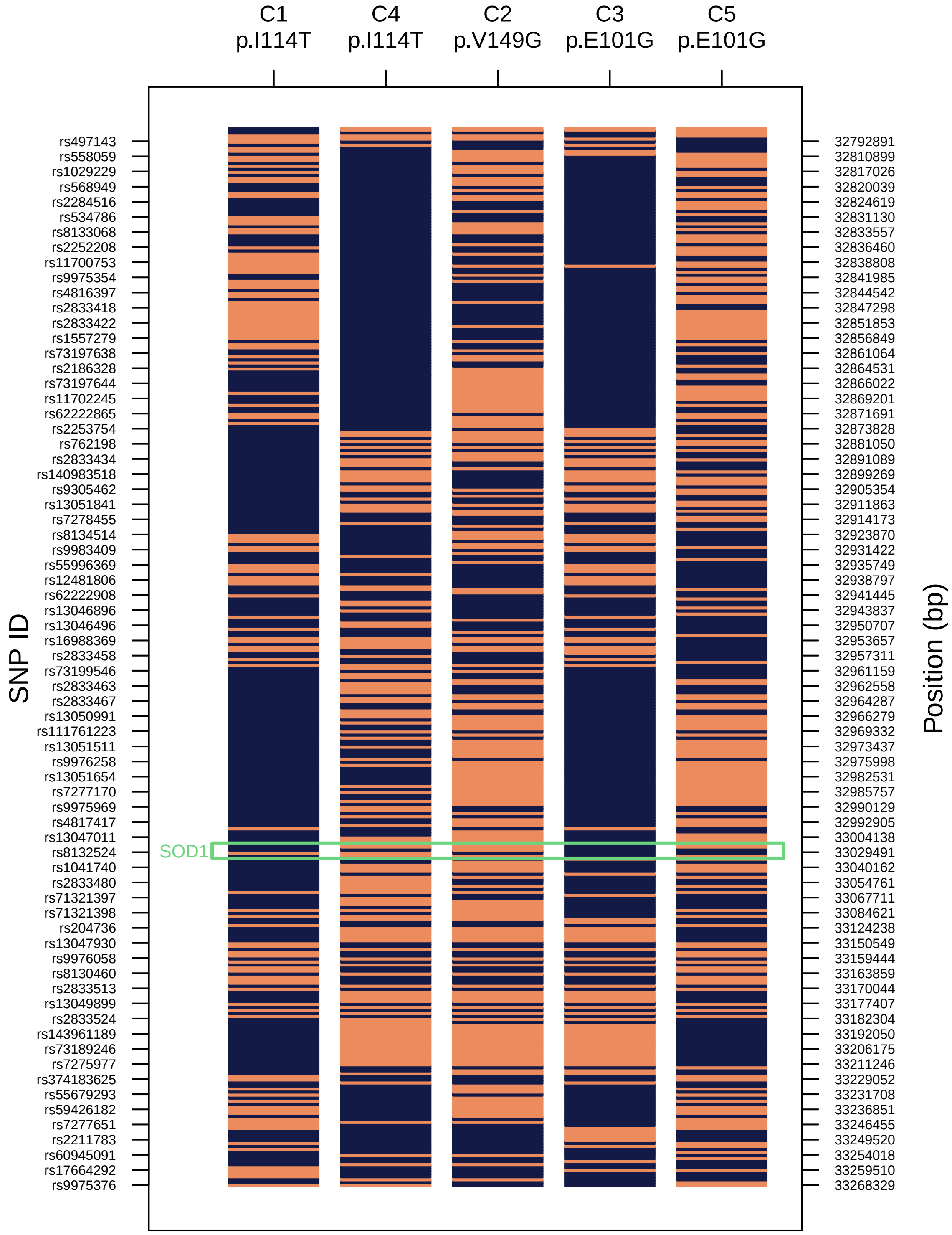

Supplementary Figure 1. Visualisation of the 350-SNP founder haplotypes for five clusters of Australian MND cases with one of three *SOD1* mutations.
Reference and alternative alleles are coloured blue and orange, respectively. SNP identifiers and their respective genomic position (hg19) are displayed for every 5th SNP beginning at SNP 1 (rs497143) in Supplementary Dataset 1.

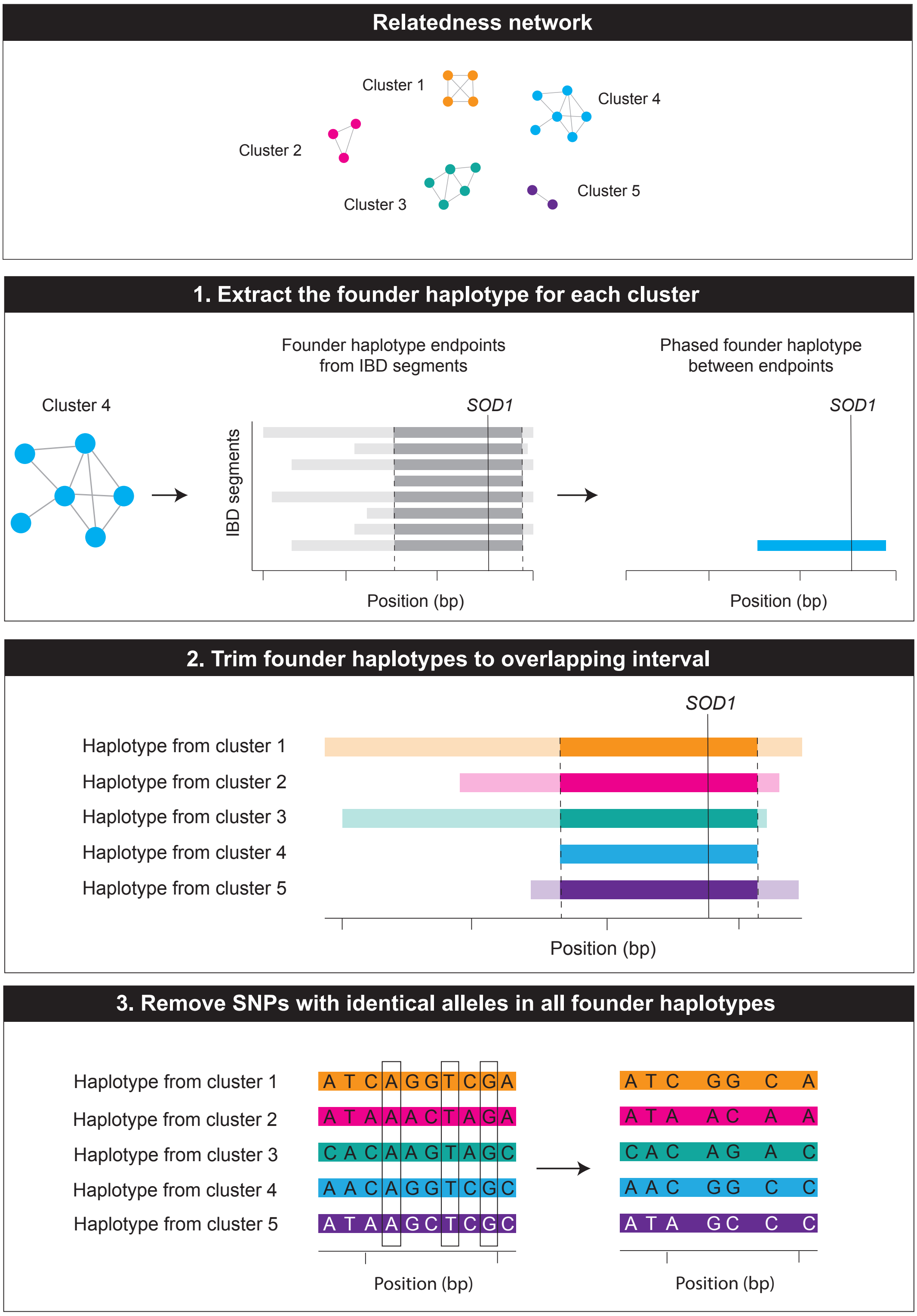

Supplementary Figure 2. The process for extracting founder haplotypes over *SOD1*. First, the founder haplotype is extracted for each cluster in the relatedness network. The endpoints of the founder haplotype for a cluster are taken as the intersection of the IBD segments inferred between all pairs of individuals within this cluster. Since the genotype data has been phased, the founder haplotype is the singular haplotype that appears in all samples within this cluster between the identified haplotype endpoints. Next, the interval that all of the founder haplotypes overlap is defined, and each founder haplotype is reported within this interval. Lastly, SNPs with an identical allele across all founder haplotypes are removed from the reported haplotypes.

Supplementary Data

Supplementary Data 1. Supplementary_data.xlsx
350-SNP founder haplotypes for five clusters of Australian MND cases with one of three *SOD1* mutations.

Supplementary Data 2. haplotype_code.R
R code to extract *SOD1* haplotypes from vcf data.

```r
# Lyndal Henden
# 13/05/2020

# extract the 5x founder haplotypes over SOD1 for 3x SOD1 mutations

# load libraries
library(SeqArray)
library(data.table)

# load IBD segments and subset by chr 21
ibd_tribes <- readRDS("path/to/ibd.match")
ibd_tribes <- ibd_tribes[ibd_tribes[,"chr"] == 21,]

# load phased WGS data for chr 21
vcf.fn <- "path/to/chr21_phased.vcf.gz"
seqVCF2GDS(vcf.fn, "path/to/chr21_phased.gds")
gds_file <- seqOpen("path/to/chr21_phased.gds")

# create a map file for chr21 from VCF with phased haplotype data
map_chr21 <- data.frame(chr=seqGetData(gds_file, "chromosome"), snp_id=seqGetData(gds_file, "annotation/id"), pos=seqGetData(gds_file, "position"), alleles=seqGetData(gds_file, "allele"),
                          allele_m1=t(seqGetData(gds_file, "genotype")[1,,]), allele_m2=t(seqGetData(gds_file, "genotype")[2,,]))
map_chr21 <- map_chr21[order(map_chr21[,"pos"]),]

# reformat phased haplotypes
sample_id <- seqGetData(gds_file, "sample.id")
allele_m1 <- map_chr21[,grep("allele_m1", colnames(map_chr21))]
allele_m2 <- map_chr21[,grep("allele_m2", colnames(map_chr21))]
map_chr21 <- map_chr21[,1:3]
phased_gt <- data.frame()
for (i in 1:ncol(allele_m1)) {
  map_chr21 <- cbind.data.frame(map_chr21, data.frame(paste(allele_m1[,i], allele_m2[,i], sep="|")))
}
colnames(map_chr21)[4:ncol(map_chr21)] <- sample_id
seqClose(gds_file)


#************************************************************
#***** get founder haplotype endpoints within a cluster *****
#************************************************************

# NOTE: this code needs to run seperately for each cluster of interest

# manually extract the sample IDs within a cluster
samples_cluster <- c("sample1", "sample2", "sample3", "sample4")

# SOD1 locus - hg19 coordinates
gene_interval <- c(21, 33031935, 33041243)
```

```
# subset IBD segments by chr 21 and samples within the cluster of interest
ibd_cluster <- ibd_tribes[ibd_tribes[,"iid1"] %in% samples_cluster & ibd_tribes[,"iid2"] %in% samples_cluster,]
ibd_cluster_chr <- ibd_cluster[ibd_cluster[,"chr"] == gene_interval[1],]

# function to extract overlap between two intervals
getOverlap <- function(region.1, region.2) {
  a <- max(region.1[1], region.2[1])
  b <- min(region.1[2], region.2[2])
  return(c(a,b))
}

# get IBD segments overlapping SOD1 for all samples within the cluster of interest
ibd_overlap <- rep(0, nrow(ibd_cluster_chr))
for(i in 1:nrow(ibd_cluster_chr)){
  my_overlap <- getOverlap(ibd_cluster_chr[i,c("start.position.bp","end.position.bp")], c(gene_interval[2], gene_interval[3]))
  if(my_overlap[2] > my_overlap[1])
    ibd_overlap[i] <- 1
}
ibd_interval <- ibd_cluster_chr[ibd_overlap == 1,]

# get genomic region common to all IBD samples over SOD1
common_region <- c(21, max(ibd_interval[,"start.position.bp"]), min(ibd_interval[,"end.position.bp"]))

# subset haplotype data for samples of interest over common region
haplotype_c1 <- map_chr21[map_chr21[,"pos"] >= common_region[2] & map_chr21[,"pos"] <= common_region[3],c("chr", "snp_id", "pos", "alleles", samples_cluster)]

# for each subsequent cluster, generate a dataframe called haplotype_c2, haplotype_c3, ..., haplotype_cn, where n is the number of clusters


#**********************************************************
#***** extract shared haplotype over common interval *****
#**********************************************************

# NOTE: this code needs to run seperately for each cluster of interest

# trim founder haplotypes to overlapping interval between all clusters - have done manually here by looking at haplotype_c1, haplotype_c2, etc
interval_overlap <- c(32792145,  33274026)

# function that extracts the most common haplotype shared between samples within a cluster over an interval
get_haplotypes <- function(haplotypes_interval) {
  haplotypes_test <- haplotypes_interval[,1:4]
  haplotypes_test_v2 <- data.frame()
  for (i in 5:ncol(haplotypes_interval)) {
    hap_i <- do.call('rbind',strsplit(as.character(haplotypes_interval[,i]), "|"))
    hap_i_df <- data.frame(hap_i[,1],hap_i[,3])
    colnames(hap_i_df) <- rep(colnames(haplotypes_interval)[i],2)
    haplotypes_test <- cbind.data.frame(haplotypes_test, hap_i_df)
```

```
    haplotypes_test_v2 <- rbind.data.frame(haplotypes_test_v2, data.frame(sample_id=colnames(haplotypes_interval)[i],hap=c(paste(hap_i_df[,1], collapse=""), paste(hap_i_df[,2], collapse=""))))
  }
  tab_hap <- table(haplotypes_test_v2[,2])

  # what is the frequency of most common haplotype
  cat(paste0("Haplotype count = ", max(tab_hap),"\n"))

  # samples with common haplotype
  cat("\nSamples with haplotype:\n")
  print(as.character(haplotypes_test_v2[haplotypes_test_v2[,2] == names(tab_hap[tab_hap == max(tab_hap)]),1]))

  # samples without common haplotype - genotype/phasing errors, ends might differ slightly
  cat("\nSamples without haplotype:\n")
  print(as.character(unique(haplotypes_test_v2[,1])[!(unique(haplotypes_test_v2[,1]) %in% as.character(haplotypes_test_v2[haplotypes_test_v2[,2] == names(tab_hap[tab_hap == max(tab_hap)]),1]))]))

  # extract haplotype over internal in data frame format
  col_same <- NULL
  for (i in 5:ncol(haplotypes_test)) {
    if (paste0(haplotypes_test[,i],collapse="") == names(tab_hap[tab_hap == max(tab_hap)]))
      col_same <- c(col_same, i)
  }

  return(list(haplotypes_test[,c(1:4,col_same)], haplotypes_test, haplotypes_test_v2, tab_hap))
}

# extract shared haplotype for a cluster
hap_cluster_1 <- haplotype_c1[haplotype_c1[,"pos"] >= interval_overlap[1] & haplotype_c1[,"pos"] <= interval_overlap[2],]
haplotypes_cluster_1 <- get_haplotypes(hap_cluster_1)
haplotype_C1 <- haplotypes_cluster_1[[1]][,c(1:5)]

# for each subsequent cluster, generate haplotypes_cluster_2, haplotype_C2 etc.


#****************************************
#***** filter and format haplotypes *****
#****************************************

# combine the shared haplotypes for each cluster into a single dataframe
haplotypes_all <- cbind.data.frame(haplotype_C1, haplotype_C4[,6], haplotype_C2[,6], haplotype_C3[,6], haplotype_C5[,6])
for (i in 6:10) haplotypes_all[,i] <- as.numeric(as.character(haplotypes_all[,i]))

# remove SNPs that are the same in all haplotypes
keep_snp <- NULL
for (i in 1:nrow(haplotypes_all)) {
  if (!all(haplotypes_all[i,6] == haplotypes_all[i,6:10]))
    keep_snp <- c(keep_snp, i)
}
haplotypes_pruned <- haplotypes_all[keep_snp,]
```

```r

# manually add 3x SOD1 variants
sod1_mut <- data.frame(chr=c(21,21,21), snp_id=c("rs121912439","rs121912441","rs1476760624"), pos=c(33039633,33039672,33040872),
                       alleles=c("A,G","T,C","T,G"), C1=c(0,1,0), C4=c(0,1,0), C2=c(1,0,0), C3=c(0,0,1), C5=c(0,0,1))
for (i in 1:3) {
  haplotypes_pruned <- rbind.data.frame(haplotypes_pruned[haplotypes_pruned[,"pos"] < sod1_mut[i,"pos"],],
                                        sod1_mut[i,],
                                        haplotypes_pruned[haplotypes_pruned[,"pos"] > sod1_mut[i,"pos"],])
}
dim(haplotypes_pruned)

# add strand and mutation status to haplotype dataframe
ref_allele <- do.call('rbind',strsplit(as.character(haplotypes_pruned[,"alleles"]),","))[,1]
alt_allele <- do.call('rbind',strsplit(as.character(haplotypes_pruned[,"alleles"]),","))[,2]
sod1_mutation <- rep(".",nrow(haplotypes_pruned))
sod1_mutation[haplotypes_pruned[,"pos"] == 33039633] <- "p.E101G"
sod1_mutation[haplotypes_pruned[,"pos"] == 33039672] <- "p.I114T"
sod1_mutation[haplotypes_pruned[,"pos"] == 33040872] <- "p.V149G"
strand <- rep("+",nrow(haplotypes_pruned))
haplotypes_save <- data.frame(haplotypes_pruned[,1:3], strand, ref_allele, alt_allele, sod1_mutation, haplotypes_pruned[,c(5:9)])
for (i in 4:7) haplotypes_save[,i] <- as.character(haplotypes_save[,i])

# change haplotypes to AGCT annotation rather than 0/1
for (i in 8:12) {
  risk_hap <- NULL
  for (j in 1:nrow(haplotypes_save)) {
    risk_hap <- c(risk_hap, ifelse(haplotypes_save[j,i] == 0, haplotypes_save[j,"ref_allele"], haplotypes_save[j,"alt_allele"]))
  }
  haplotypes_save[,i] <- risk_hap
}
colnames(haplotypes_save) <- c("Chromosome", "SNP ID", "Position (hg19)", "Strand", "Ref", "Alt", "SOD1 Mutation",
                               "C1 - p.I114T", "C4 - p.I114T", "C2 - p.V149G", "C3 - p.E101G", "C5 - p.E101G")


#*****************************************************
#***** add 1000K EUR + gnomAD allele frequencies *****
#*****************************************************

#*** 1000K ***

# read in phased 1000 genomes chr 21 VCF file - downloaded from: ftp://ftp.1000genomes.ebi.ac.uk/vol1/ftp/release/20130502/
vcf.fn <- "path/to/ALL.chr21.phase3_shapeit2_mvncall_integrated_v5a.20130502.genotype.vcf.gz"
seqVCF2GDS(vcf.fn, "path/to/ALL.chr21.phase3_shapeit2_mvncall_integrated_v5a.20130502.genotype.gds")
gds_file <- seqOpen("path/to/ALL.chr21.phase3_shapeit2_mvncall_integrated_v5a.20130502.genotype.gds")
seqSetFilter(gds_file)

```

```r
# subset by variants in haplotypes
snp_id <- seqGetData(gds_file, "annotation/id")
variant_id <- seqGetData(gds_file, "variant.id")
seqSetFilter(gds_file, variant.id=variant_id[snp_id %in% haplotypes_save[,"SNP ID"]])

# subset allele freq
ceu_freq <- seqGetData(gds_file, "annotation/info/EUR_AF")

# get alleles
alleles_1k <- seqGetData(gds_file, "allele")
ref_1k <- do.call('rbind', strsplit(alleles_1k, ","))[,1]
alt_1k <- do.call('rbind', strsplit(alleles_1k, ","))[,2]

# get alleles
map_1k <- data.frame(chr=seqGetData(gds_file, "chromosome"), snp_id=seqGetData(gds_file, "annotation/id"), pos=seqGetData(gds_file, "position"),
                     REF=ref_1k, ALT=alt_1k, ceu_freq[[2]])
seqClose(gds_file)

#*** gnomad ***

# read in allele frequencies manually collated for haplotype SNPs from webpage
gnomad <- fread("path/to/haplotype_coords_gnomAD_anno.csv", data.table=FALSE)

# merge with 1000 genomes map file - 3 SOD1 mutations missing from 1k data, 2 missing from gnomad
gnomad_1k <- merge(map_1k, gnomad, by.x=c("snp_id","REF","ALT"), by.y=c("snp","Reference","Alternate"))
gnomad_1k <- gnomad_1k[order(gnomad_1k[,"pos.x"]),]

# look at difference in allele freq between 1k and gnomad
hist(abs(gnomad_1k[,"ceu_freq..2.."] - gnomad_1k[,"AF"]), xlim=c(0,1), xlab="diff", main="")
# looks great!

# create new haplotype file with updated allele frequencies
haplotypes_save$freq_gnomad <- 0
haplotypes_save$freq_ceu <- 0
for (i in 1:nrow(haplotypes_save)) {
  if (haplotypes_save[i,"SNP ID"] %in% gnomad_1k[,"snp_id"]) {
    haplotypes_save[i,"freq_ceu"] <- gnomad_1k[gnomad_1k[,"snp_id"] %in% haplotypes_save[i,"SNP ID"],"ceu_freq..2.."]
    haplotypes_save[i,"freq_gnomad"] <- gnomad_1k[gnomad_1k[,"snp_id"] %in% haplotypes_save[i,"SNP ID"],"AF"]
  }
}
haplotypes_save_v2 <- haplotypes_save[,c(1:3,13,14,4:12)]
colnames(haplotypes_save_v2)[4:5] <- c("Reference Allele Freq - 1000 Genomes EUR", "Reference Allele Freq - gnomAD non-neuro non-Finnish EUR")

# change SOD1 allele freq
haplotypes_save_v2[haplotypes_save_v2[,"SNP ID"] %in% c("rs121912439","rs121912441","rs1476760624"),"Reference Allele Freq - 1000 Genomes EUR"] <- NA
haplotypes_save_v2[haplotypes_save_v2[,"SNP ID"] %in% c("rs121912439","
```

```
rs121912441","rs1476760624"),"Reference Allele Freq - gnomAD non-neuro non-Finnish EUR"] <- NA
haplotypes_save_v2[haplotypes_save_v2[,"SNP ID"] %in% "rs121912441","Reference Allele Freq - gnomAD non-neuro non-Finnish EUR"] <- 0.0000111664

# look at haplotypes
haplotypes_save_v2
```
