# Supplementary material for: Identity by descent analysis identifies founder events and links SOD1 familial and sporadic ALS cases
Source: NPJ Genom Med. 2020 Aug 7;5:32. doi: 10.1038/s41525-020-00139-8 (PMC7414871; doi:10.1038/s41525-020-00139-8)
Supplement: Supplementary file 3 — Reporting Summary [file 41525_2020_139_MOESM3_ESM.pdf]

nature research

Corresponding author(s): Kelly Williams

Last updated by author(s): 25/06/2020

Reporting Summary

Nature Research wishes to improve the reproducibility of the work that we publish. This form provides structure for consistency and transparency in reporting. For further information on Nature Research policies, see our Editorial Policies and the Editorial Policy Checklist.

Statistics

For all statistical analyses, confirm that the following items are present in the figure legend, table legend, main text, or Methods section.

n/a	Confirmed	
☐	☒	The exact sample size (*n*) for each experimental group/condition, given as a discrete number and unit of measurement
☒	☐	A statement on whether measurements were taken from distinct samples or whether the same sample was measured repeatedly
☒	☐	The statistical test(s) used AND whether they are one- or two-sided<br>*Only common tests should be described solely by name; describe more complex techniques in the Methods section.*
☒	☐	A description of all covariates tested
☒	☐	A description of any assumptions or corrections, such as tests of normality and adjustment for multiple comparisons
☐	☒	A full description of the statistical parameters including central tendency (e.g. means) or other basic estimates (e.g. regression coefficient) AND variation (e.g. standard deviation) or associated estimates of uncertainty (e.g. confidence intervals)
☒	☐	For null hypothesis testing, the test statistic (e.g. *F*, *t*, *r*) with confidence intervals, effect sizes, degrees of freedom and *P* value noted<br>*Give P values as exact values whenever suitable.*
☒	☐	For Bayesian analysis, information on the choice of priors and Markov chain Monte Carlo settings
☒	☐	For hierarchical and complex designs, identification of the appropriate level for tests and full reporting of outcomes
☒	☐	Estimates of effect sizes (e.g. Cohen's *d*, Pearson's *r*), indicating how they were calculated

Our web collection on statistics for biologists contains articles on many of the points above.

Software and code

Policy information about availability of computer code

Data collection	No software used
Data analysis	bcl2fastq v2.16.0; BWA-MEM v0.7.15-r1140; bamsormadup v2.0.65; GATK v3.7-0gcfedb67; ANNOVAR; VeirfyBamID v1.1.3; TRIBES; isoRelate; The Gamma method

For manuscripts utilizing custom algorithms or software that are central to the research but not yet described in published literature, software must be made available to editors and reviewers. We strongly encourage code deposition in a community repository (e.g. GitHub). See the Nature Research guidelines for submitting code & software for further information.

Data

Policy information about availability of data

All manuscripts must include a data availability statement. This statement should provide the following information, where applicable:

- Accession codes, unique identifiers, or web links for publicly available datasets
- A list of figures that have associated raw data
- A description of any restrictions on data availability

The data that support the findings of this study are available on reasonable request from the corresponding author [KW]. The data are not publicly available since our ethics permission does not cover sharing of data to third parties.

Field-specific reporting

Please select the one below that is the best fit for your research. If you are not sure, read the appropriate sections before making your selection.

☒ Life sciences ☐ Behavioural & social sciences ☐ Ecological, evolutionary & environmental sciences

For a reference copy of the document with all sections, see nature.com/documents/nr-reporting-summary-flat.pdf

Life sciences study design

All studies must disclose on these points even when the disclosure is negative.

Sample size	86/850 Australian ALS cases with WGS data carry a SOD1 mutation and were selected for inclusion in this manuscript.
Data exclusions	All samples with WGS data that did not carry a SOD1 mutation were excluded from the analysis as we sought to identify common founders between SOD1 mutation carriers only.
Replication	Results were replicated in an independent analysis of the same cohort using a second IBD methodology.
Randomization	Randomization is not relevant to this study as subgroups were defined according to SOD1 mutation status, which defined 3 independent subgroups.
Blinding	Blinding is not relevant to this study as we required knowledge of mutation subgroup status.

Reporting for specific materials, systems and methods

We require information from authors about some types of materials, experimental systems and methods used in many studies. Here, indicate whether each material, system or method listed is relevant to your study. If you are not sure if a list item applies to your research, read the appropriate section before selecting a response.

Materials & experimental systems

n/a	Involved in the study	
☒	☐	Antibodies
☒	☐	Eukaryotic cell lines
☒	☐	Palaeontology and archaeology
☒	☐	Animals and other organisms
☐	☒	Human research participants
☒	☐	Clinical data
☒	☐	Dual use research of concern

Methods

n/a	Involved in the study	
☒	☐	ChIP-seq
☒	☐	Flow cytometry
☒	☐	MRI-based neuroimaging

Human research participants

Policy information about studies involving human research participants

Population characteristics	86 Australian ALS cases carrying one of three SOD1 mutations (p.I114T, p.E101G, p.V149G). 72% of cases are affected, while the remaining 28% are asymptomatic SOD1 mutation carries. The age at disease onset varies between 25 and 76 years old with an even distribution of males and females.
Recruitment	Participants were recruited for analysis from the Macquarie University Neurodegenerative Disease Biobank, Molecular Medicine Laboratory (Concord Hospital), Australian MND DNA Bank (Royal Prince Alfred Hospital) and Brain and Mind Centre (University of Sydney).
Ethics oversight	Informed written consent was approved by the human research ethics committees of the Sydney South West Area Health Service, Macquarie University (5201600387), or University of Sydney.

Note that full information on the approval of the study protocol must also be provided in the manuscript.
